# Andrographolide Derivative AL-1 Ameliorates Dextran Sodium Sulfate-Induced Murine Colitis by Inhibiting NF-*κ*B and MAPK Signaling Pathways

**DOI:** 10.1155/2019/6138723

**Published:** 2019-10-07

**Authors:** Mei Jing, Yuqiang Wang, Lipeng Xu

**Affiliations:** Institute of New Drug Research, Guangzhou Key Laboratory of Innovative Chemical, Drug Research in Cardio-Cerebrovascular Diseases and International Cooperative Laboratory of Traditional Chinese Medicine Modernization and Innovative Drug Development of Chinese Ministry of Education (MOE), Jinan University College of Pharmacy, Guangzhou 510632, China

## Abstract

Trinitrobenzenesulfonic acid (TNBS) and dextran sodium sulfate (DSS) are commonly used to induce experimental murine ulcerative colitis (UC). Our recent study has demonstrated that a novel andrographolide derivative, AL-1, ameliorated TNBS-induced colitis in mice. However, the effect of AL-1 on DSS-induced murine colitis and the underlying mechanisms are yet unknown. In the present study, we aimed to investigate the therapeutic potential of AL-1 against DSS-induced UC in mice and to define its mechanisms of action. Oral administration of AL-1 attenuated body weight loss, reduced colon length shortening, lowered the disease activity index score, and alleviated colon histological damage. AL-1 significantly inhibited myeloperoxidase activity and suppressed immune inflammatory responses in colonic tissues. Moreover, AL-1 reversed DSS-altered expression of inflammatory cytokines in DSS-induced colitis mice. Importantly, the efficacy of 45 mg/kg of AL-1 was higher than that of 100 mg/kg of the positive control drugs 5-aminosalicylic acid and mesalazine. AL-1 decreased lipopolysaccharide-induced generation of reactive oxygen species and nitric oxide in cultured macrophages in vitro; it also reversed the altered expression of inflammatory cytokines. In both in vivo and in vitro studies, Western blot analysis revealed that AL-1 reduced the expression of phosphorylated NF-*κ*B p65 and I*κ*B*α*, downregulated the expression of iNOS and COX-2, and attenuated the expression of phosphorylated p38 mitogen-activated protein kinase (MAPK), ERK, and JNK. In conclusion, AL-1 alleviated DSS-induced murine colitis by inhibiting activation of the NF-*κ*B and MAPK signaling pathways. Our data suggest that AL-1 could be a potential new treatment for UC.

## 1. Introduction

Ulcerative colitis (UC) is a chronic inflammatory disorder of the colon characterized by idiopathic, chronic, relapsing, and inflammatory conditions in the gastrointestinal tract [1, 2]. It has a high prevalence worldwide and is a well-established risk factor for colorectal cancer [3]. The primary goals of therapy in the treatment of UC are to induce remission of patient symptoms as rapidly as possible and maintain remission on a long-term basis. Steroid hormones, immunosuppressive agents, or their derivatives are used to treat UC with modest results and often with serious side effects. New therapies need to be developed to increase the number and duration of remissions. Currently, 5-aminosalicylates (5-ASAs) are the standard treatment for the induction and maintenance of remission in mild-to-moderate ulcerative colitis patients. However, the majority of UC patients present with moderate-to-severe disease (80%) rather than mild disease (20%). Additional treatment options other than 5-ASAs are considered for patients or those who do not respond to 5-ASA. Despite extensive research conducted over many years, the precise etiology of UC remains unclear. However, increasing experimental and clinical evidences suggest that the increase of proinflammatory cytokines such as interleukin-1*β* (IL-1*β*) and tumor necrosis factor-*α* (TNF-*α*) in colonic tissues plays a pivotal role in the pathogenesis of UC [4, 5]. Therefore, inhibiting or downregulating these inflammatory mediators or their upstream regulators, such as NF-*κ*B p65, may offer an alternative therapy for UC [6, 7].

NF-*κ*B comprises a family of inducible transcription factors that serve as important regulators of the inflammatory response [8, 9]. In its inactivated state, NF-*κ*B combines with the inhibitory cytoplasmic protein I*κ*B. Once activated, the p50/p65 subunits of NF-*κ*B are transferred into the nucleus to boost the expression of inflammatory proteins, resulting in inflammation and oxidative stress [8, 10]. Persistent activation of NF-*κ*B signaling has been detected in the mucosa of inflammatory bowel disease (IBD) patients and in murine IBD models [11, 12]. Subsequently, the p50/p65 complex translocates to the nucleus and binds to specific binding sites in the promoter regions of its target genes including TNF-*α*, inducible nitric oxide synthase (iNOS), cyclooxygenase-2 (COX-2), and chemokines. Inhibition of NF-*κ*B with a specific p65 antisense oligonucleotide has been reported to have benefits in IBD experimental mouse models [13]. Increased MAPK activation has also been revealed to increase the development of IBD due to the induction of iNOS expression [14]. Accordingly, targeting the NF-*κ*B and MAPK signaling pathways is considered an attractive therapeutic strategy for managing intestinal inflammation.

**Figure 1:**
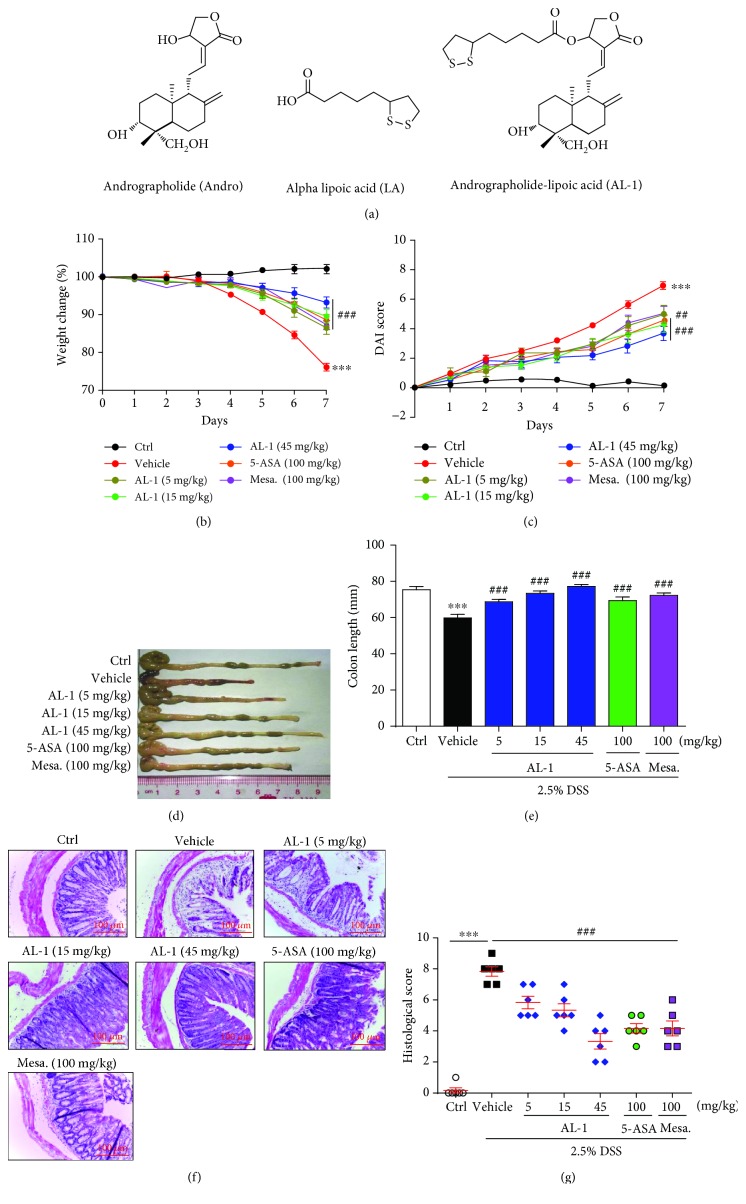
AL-1 attenuates colitis in the DSS-induced mice model. (a) The chemical structure of AL-1. (b) Body weight change and (c) DAI scores of C57BL/C mice given 2.5% DSS for 7 days. Mice were sacrificed on day 8, and colon length was measured (d, e). H&E-stained colon cross-sections of C57BL/C mice at day 8 (f). Total histological score was calculated as the sum of epithelial damage (g). Data are presented as the mean ± SEM (*n* = 6‐17 mice/group). ^∗∗∗^*P* < 0.001 compared with the control group and ^##^*P* < 0.01 and ^###^*P* < 0.001 compared with the vehicle-treated UC model group.

Andrographolide (Andro, Figure 1(a)) is the major active compound of the medicinal plant *Andrographis paniculata*. Andro has been extensively used in traditional herbal medicine in China, Thailand, India, and other Asian countries for the treatment of several diseases, including IBD [15–19]. Lipoic acid (LA, Figure 1(a)) is a multifunctional antioxidant effective in ameliorating symptoms of diseases associated with oxidative stress [20]. LA also possesses an anti-inflammatory effect independent of its antioxidant activity [21, 22]. An earlier study illustrated that LA attenuates LPS-induced inflammatory responses in human monocyte cells as well as in rodent lung, heart, and aorta tissues, suggesting that LA can play a role in sepsis and inflammatory diseases [23]. It has been reported that LA can inhibit both adhesion molecule expression in human aortic endothelial cells and monocyte adhesion by inhibiting the NF-*κ*B signaling pathway [24].

We previously designed and synthesized a novel Andro derivative AL-1 (Figure 1(a)) by covalently linking Andro with LA [25]. In our previous study, we have demonstrated that the anti-inflammatory and/or antioxidative activity of AL-1 contributed to its cytoprotective effects and AL-1 ameliorated trinitrobenzenesulfonic acid- (TNBS-) induced colitis in mice [26–28]. However, whether AL-1 can ameliorate dextran sodium sulfate- (DSS-) induced murine colitis and the mechanisms responsible for its action has not yet been explored. Therefore, in this study, we investigated the effects of AL-1 on DSS-induced colitis in C57BL/6 mice and upon lipopolysaccharide- (LPS-) induced macrophages in vitro and elucidated the possible role of NF-*κ*B and MAPK pathways in AL-1's underlying mechanisms of action.

## 2. Methods

### 2.1. Animals

Male and female C57BL/6 mice 6-8 weeks of age were purchased from the Laboratory of Animal Center of Sun Yat-sen University (certificate SYXK 2011-0112, Guangzhou, China). The animals were housed in plastic cages, four mice per cage, at a constant temperature (23°C) and maintained in air-conditioned quarters with 12 h light/dark cycles. The animals were acclimatized in the laboratory for 1 week before use. All animal welfare and experimental procedures were in accordance with and approved by the Research Ethics Committee of Jinan University.

### 2.2. Experimental Procedures

Animals were randomly assigned into seven groups: normal control group, UC model group, AL-1 low-dose group (5 mg/kg), AL-1 middle-dose group (15 mg/kg), AL-1 high-dose group (45 mg/kg), 5-ASA group (100 mg/kg), and mesalazine group (100 mg/kg). With the exception of the normal control group (which was given tap water without DSS to drink), acute colitis was induced in all other groups by administration of 2.5% (wt/vol) DSS (molecular weight 36-50 kDa) in tap water ad libitum for 7 days. AL-1, 5-ASA, and mesalazine were given by oral gavage twice a day from day 1 to day 7 on 7-9 weeks of age. The 5-ASA and mesalazine were used as positive control drugs. The animals were sacrificed on day 8, and the colons (from the caecum to the anus) were removed.

### 2.3. Disease Activity Index (DAI) and Histological Observation

DAI and histological scoring was assessed by three investigators blinded to the protocol according to a standard scoring system. The DAI is widely used for colitis, which scores the severity of the disease. Body weight, stool consistency, and the presence of gross blood in feces were assessed daily for each mouse. These parameters were each assigned a score as reported previously [29, 30]. Loss in body weight was scored as follows: 0, no weight loss; 1, weight loss of 1-5%; 2, weight loss of 5-10%; 3, weight loss of 10-20%; and 4, more than 20% weight loss. Stool consistency was used for calculation as follows: 0, normal stool pellets; 2, unformed stool; and 4, watery diarrhea. Occult blood was measured and graded as follows: 0, no rectal bleeding or blood in stool; 2, weak haemoccult-positive spots in stool; and 4, fresh rectal bleeding. The DAI was calculated as the total of these scores. Subsequently, the distal colonic tissues for histopathology were fixed into 4% paraformaldehyde solution, embedded in paraffin, and sectioned at 5 *μ*m thickness sections to determine the degree of inflammation [31]. Histological scores were blindly determined according to the standard scoring system [3, 29]. The histological scoring system was used as follows: (i) crypt damage—0, none; 1, basal 1/3 damage; 2, basal 2/3 damage; 3, crypts lost and surface epithelium present; and 4, crypts and surface epithelium lost; (ii) infiltration of mononuclear and polymorphonuclear cells—0, none; 1, crypt basis; 2, mucosa; 3, mucosa and submucosa; and 4, transmural; and (iii) erosion ulceration—0, intact epithelium; 2, areas of ulceration involving the submucosa; and 4, areas of ulceration involving the transmural. Total histological score was calculated by summation of the scores of crypt damage, infiltration of mononuclear and polymorphonuclear cells, and erosion ulceration.

### 2.4. Measurement of Myeloperoxidase (MPO) Activity of Colonic Tissue

To measure MPO activity, the imaging system IVIS 100 (Xenogen Corp., Alameda, CA, USA) was used, which consists of a light tight chamber equipped with a cooled CCD camera. The luminescent probe L-012 (8-amino-5-chloro-7-phenylpyrido [3, 4-*d*] pyridazine-1, 4 (2H, 3H) dione sodium salt) (Wako Chemical) was freshly dissolved in sterile H_2_O at a concentration of 20 mM. Animals were intraperitoneally treated with 100 *μ*L of L-012 [32]. During in vivo imaging, the mice were anesthetized with isoflurane (1.5%). The mice were euthanized for subsequent collection of the colon tissue. Ex vivo imaging was done on the dissected organs using the IVIS 100 system. Tissue samples were obtained from all groups, and neutrophil infiltration into inflamed colonic mucosa was quantified by MPO activity assessment. The level of MPO in colon tissue was studied according to the manufacturer's instructions. The values were recorded as activity units per mg of tissue.

### 2.5. Immunohistochemistry (IHC)

To evaluate the severity of colitis on day 8, the anti-Gr-1 antibody, anti-CD3 antibody, and anti-Mac-2 antibody were used for IHC staining. For IHC evaluation, paraffin-embedded sections (4 *μ*m) of distal colonic tissues were analyzed for neutrophils (Gr-1-positive cells), T-lymphocytes (CD3-positive cells), and macrophages (Mac-2-positive cells). Slices were incubated overnight at 4°C with anti-rabbit Gr-1 antibody, anti-rabbit CD3 antibody, and anti-rabbit Mac-2 antibody. Biotin-labeled secondary antibody and streptavidin-HRP were incubated for 40 min at room temperature. Immunoreactions were analyzed with a light microscope (Olympus, Japan). 10 images of Gr-1-, CD3-, and Mac-2-stained colon sections for each mouse were counted (original magnification: ×200) [33].

### 2.6. Cell Culture

RAW 264.7 cells were purchased from the American Type Culture Collection (ATCC, Rockville, MD, USA). The cells were cultured in Dulbecco's modified Eagle's medium (DMEM) supplemented with 10% fetal bovine serum under a humidified 5% (*v*/*v*) CO_2_ atmosphere at 37°C.

### 2.7. Determination of Intracellular Formation of ROS and NO

The RAW 264.7 cells (2 × 10^4^ cells/well) were plated in fluorescence microtiter 96-well plates. The cells were treated with LPS (1 *μ*g/mL) and different concentrations of AL-1 for 24 h. Intracellular ROS level was evaluated using a radical sensitive DCFH-DA fluorescent probe [34]. The cells were incubated with 20 *μ*M DCF-DA for 30 min at 37°C in darkness. The cells were washed with HBSS for three times. DCF fluorescence was measured at 485/20 nm excitation and 528/20 nm emission in a BioTek Synergy HT microplate reader or fluorescence microscope (Olympus, Japan) [34]. In this experiment, 3-amino,4-aminomethyl-2′,7′-difluorescein, diacetate (DAF-FM DA) was used as a fluorescent indicator of intracellular NO. After loading with 5 *μ*M DAF-FM DA (Beyotime Institute of Biotechnology, China) at 37°C for 20 min in darkness, the RAW 264.7 cells were gently washed three times with HBSS. After washing out the excess probe, the cells were maintained in HBSS throughout the experiments. DAF-FM fluorescence was detected at an excitation wavelength of 485 nm and an emission wavelength of 528 nm with a BioTek Synergy HT microplate reader or fluorescence microscope (Olympus, Japan).

### 2.8. Enzyme-Linked Immunoassay (ELISA)

The levels of IL-1*β*, IL-6, TNF-*α*, IFN-*γ*, PGE_2_, and IL-10 in the mouse sera were measured using commercially available ELISA kits (Nanjing Jiancheng Bioengineering Institute, Nanjing, China). The IL-1*β*, IL-6, TNF-*α*, and PGE_2_ productions in the supernatant of RAW 264.7 cells were determined using ELISA kits (R&D Systems, Minneapolis, MN, USA) as per the manufacturer's recommendations.

### 2.9. Immunofluorescence Cytochemistry

The RAW 264.7 cells were plated on glass bottom dishes at a density of 2 × 10^4^ cells/well. After drug treatment, cells were fixed for 10 min by 4% paraformaldehyde. Cells were permeabilized with 0.5% Triton X-100 for 10 min at RT [35]. The cells were blocked with 10% horse serum for 30 min and then immunostained with an anti-NF-*κ*B p65 primary antibody overnight at 4°C [36]. After washing out the excess primary antibody, cells were treated with Alexa Fluor 647-confugated secondary antibody (Cell Signaling Technology, Boston, MA) and DAPI (Thermo Scientific, Rockford, IL) 1 h in the dark. The fluorescence was visualized under a laser confocal microscope (Carl Zeiss, Germany).

### 2.10. Quantitative Real-Time Polymerase Chain Reaction (qPCR)

Total RNAs were isolated using the RNAiso Plus reagent (Takara Bio Inc., Shiga, Japan) from the RAW 264.7 cells. Complementary DNA was synthesized from 1 *μ*g of total RNA using reverse transcriptase (Takara Bio Inc., Shiga, Japan). The sequences of specific primers used are shown in Table 1. Gene expression was quantified using SYBR® Premix Ex Taq™ II (Takara Bio Inc., Shiga, Japan) in a CFX96TM Real-Time PCR Detection System. The mRNA gene expression levels were normalized to *β*-actin gene expression levels.

### 2.11. Western Blotting

Colon tissues were homogenized with RIPA buffer containing 1 mM PMSF (Sigma-Aldrich, St. Louis, MO) and 1% phosphatase inhibitors (Beyotime Institute of Biotechnology, China). The precipitate was removed by centrifugation at 12000 g for 30 min at 4°C. The total protein concentration of the supernatant was determined using a BCA protein assay kit (Fdbio Science, China). The samples were separated on 10% SDS-PAGE gel and transferred to PVDF membranes (Millipore; Billerica, MA, USA) [37]. The membranes were blocked with 5% skim milk for 1 h at room temperature. Following standard methods to incubate the membrane with appropriate primary antibodies, the primary antibodies included those that selectively recognize p65, p-p65, p-I*κ*B*α*, I*κ*B*α*, p-p38, p38, p-ERK, ERK, p-JNK, JNK, iNOS, COX-2, and TNF-*α* (Cell Signaling Technology, Danvers, MA, USA). Target proteins were detected using corresponding HRP-conjugated anti-rabbit IgG and anti-mouse IgG, as secondary antibodies. The signals were visualized using an ECL Western blot detection kit (Fdbio Science, China). The results were captured and quantified using the Carestream Molecular Imaging system (Carestream Health, Inc., USA).

### 2.12. Statistical Analysis

Data analyses were performed with the SPSS software, version 17.0 (SPSS Inc., Chicago, IL, USA). The experimental data were expressed as the mean ± SEM. One-way analysis of variance (ANOVA) followed by a least significant difference (LSD) test was used to make comparisons among the groups. *P* < 0.05 was considered statistically significant.

## 3. Results

### 3.1. AL-1 Attenuates DSS-Induced Experimental Colitis

It is well known that DSS induces a severe illness in mice characterized by a dramatic loss of body weight, significant appearance of diarrhea/loose feces, and visible fecal blood as evaluated by DAI. Compared with animals in the vehicle-treated model group, AL-1 at concentrations of 5, 15, and 45 mg/kg significantly attenuated the loss of body weight and elevation of DAI throughout the disease progression (Figures 1(b) and 1(c)). DSS typically caused colonic shortening, and such change was also improved by treatments with AL-1 at 5, 15, and 45 mg/kg (Figures 1(d) and 1(e)). Mucosal damage, which is characterized by ulceration with massive infiltration of granulocytes and mononuclear cells into the mucosa as well as congestion and edema of the submucosa, was readily observed in the DSS-treated mice. Histologic analysis by H&E staining was used to verify the protective effect of AL-1 against colitis. AL-1 helped mice to maintain intact colonic architecture with no obvious inflammatory cell infiltration or ulceration (Figure 1(f)). Administration of AL-1 significantly reduced the severity scores of colitis-induced injury (Figure 1(g)). All the changes in DSS-induced colitis were also alleviated by treatment with 100 mg/kg of the positive control drugs mesalazine or 5-ASA.

### 3.2. AL-1 Suppresses Inflammatory Responses Induced by DSS Administration

**Figure 2:**
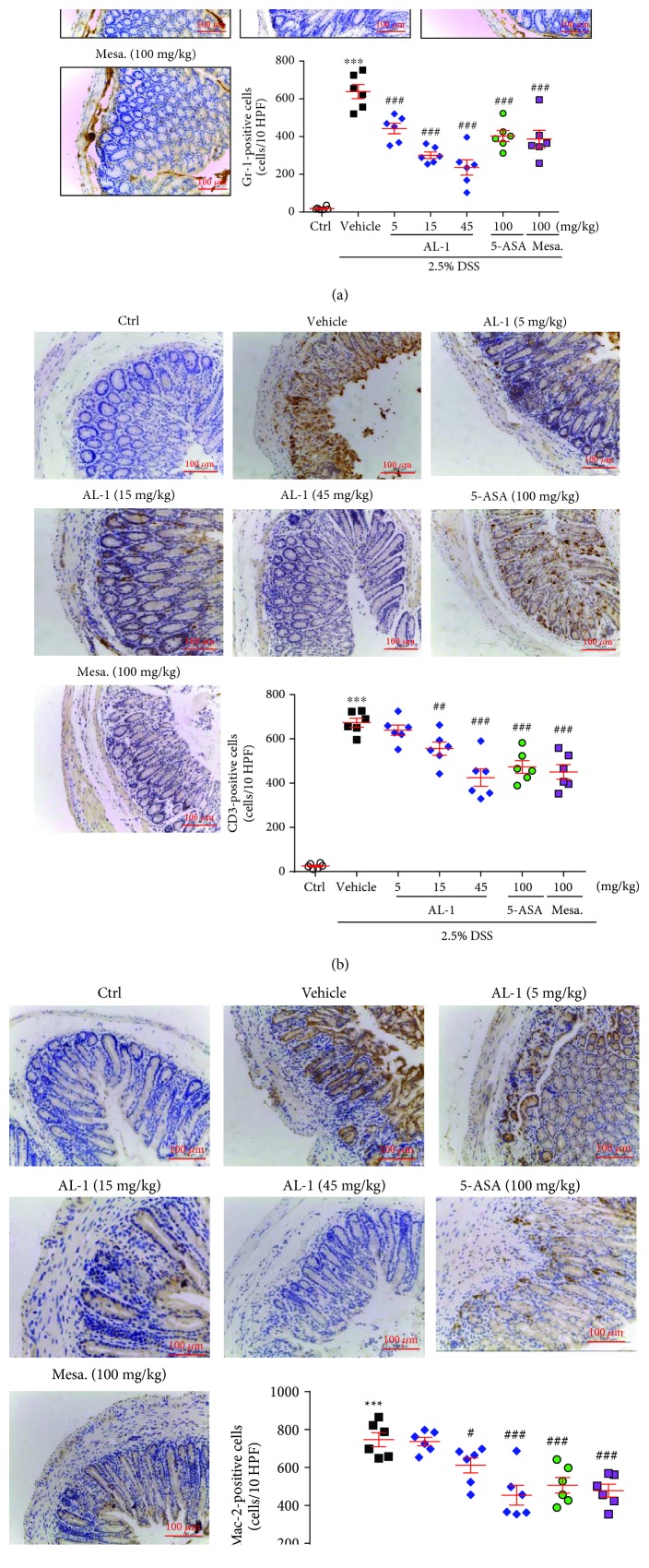
AL-1 suppresses the inflammatory responses in acute colitis after DSS administration. (a) IHC for Gr-1 showing the numbers of accumulated neutrophils in the acutely injured mouse colonic mucosa. (b) IHC for CD3 showing infiltration of T-lymphocytes, especially around the crypts in the lamina propria. (c) IHC for Mac-2 showing the number of perimucosal macrophages in the mice. Scale bar = 100 *μ*m. Data are presented as the mean ± SEM (*n* = 6 mice/group, 3 male and 3 female mice). ^∗∗∗^*P* < 0.001 compared with the control group and ^#^*P* < 0.05, ^##^*P* < 0.01, and ^###^*P* < 0.001 compared with the vehicle-treated UC model group.

To further define the inflammatory response induced by DSS administration, paraffin sections of colonic tissue were stained with anti-Gr-1, anti-CD3, and anti-Mac-2 antibodies (Figure 2). No or few Gr-1-positive neutrophils, CD3-positive T-lymphocytes, and Mac-2-positive macrophages were observed in the normal control mice (Figure 2(a)). In contrast, the colonic tissues in the DSS+vehicle group contained large numbers of Gr-1, CD3, and Mac-2 immunoreactive cells, especially around the crypts in the lamina propria and perimucosal regions. Treatment with AL-1, mesalazine, or 5-ASA all significantly suppressed the immune inflammatory cell response (Figure 2).

### 3.3. AL-1 Reduces MPO Activity in the DSS-Induced Colitis Mice

**Figure 3:**
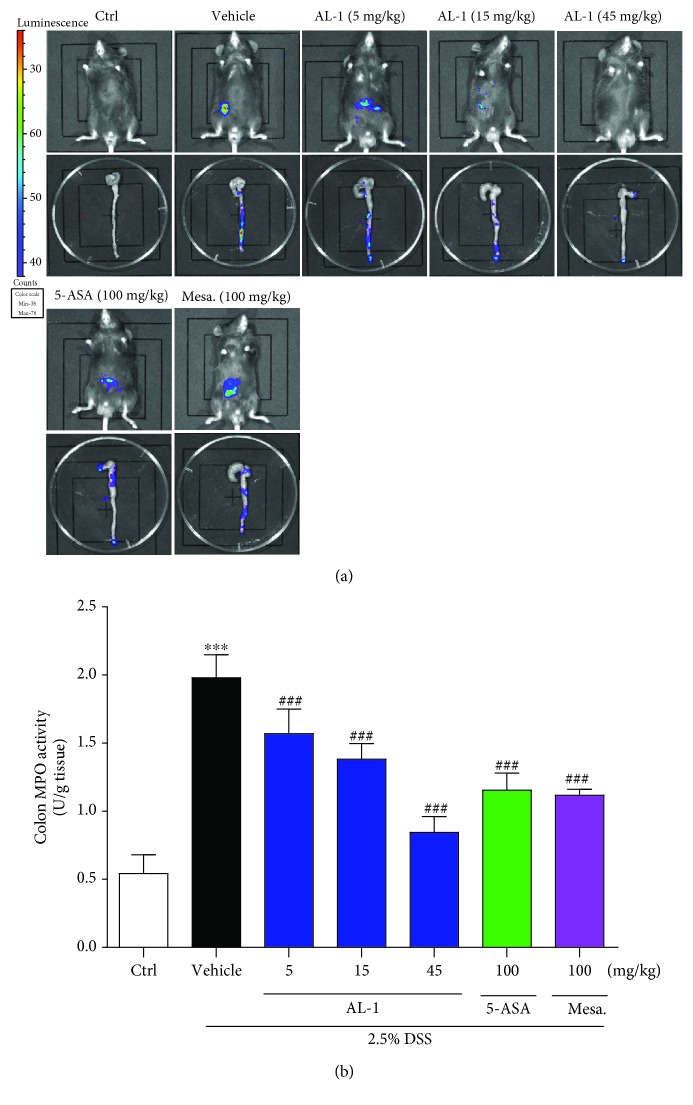
AL-1 suppresses recruitment of immune inflammatory cells. (a) In vivo MPO activity in live mice and ex vivo MPO activity in dissected organs by imaging of luminescence. (b) Protein from colonic tissue was extracted, and MPO level was determined. Data are presented as the mean ± SEM (*n* = 6 male mice/group). ^∗∗∗^*P* < 0.001 compared with the control group and ^###^*P* < 0.001 compared with the vehicle-treated UC model group.

MPO activity, a biochemical maker for neutrophil influx, was assessed to evaluate the potency of AL-1 at reducing inflammatory cell infiltration. The chemiluminescent probe L-012, an analogue of luminol, was used as a marker; upon exposure to an oxidizing agent, L-012 emits blue luminescence [38]. We observed particularly high luminescence from the abdominal area in the DSS-induced colitis mice on day 8. In contrast, mice in the control group did not exhibit any luminescence (Figure 3(a)). To elucidate the origin of this MPO signal, we dissected colonic tissues and imaged them ex vivo. As shown in Figure 3(a), a distinct light signal was observed from the distal part of the small intestine. In comparison to the DSS+vehicle-treated group, we observed that AL-1 treatment resulted in a gradual decrease in photoemission over the abdominal area and dissected colonic tissue in a dose-dependent fashion. Moreover, AL-1 treatment significantly attenuated the activity of MPO (Figure 3(b)) in the mouse colonic tissue. Treatment with the positive control drugs mesalazine or 5-ASA predictably also attenuated the elevated MPO signal and MPO activity induced by DSS.

### 3.4. AL-1 Regulates the Altered Cytokine Profiles in the Sera of Mice with DSS-Induced Colitis

**Figure 4:**
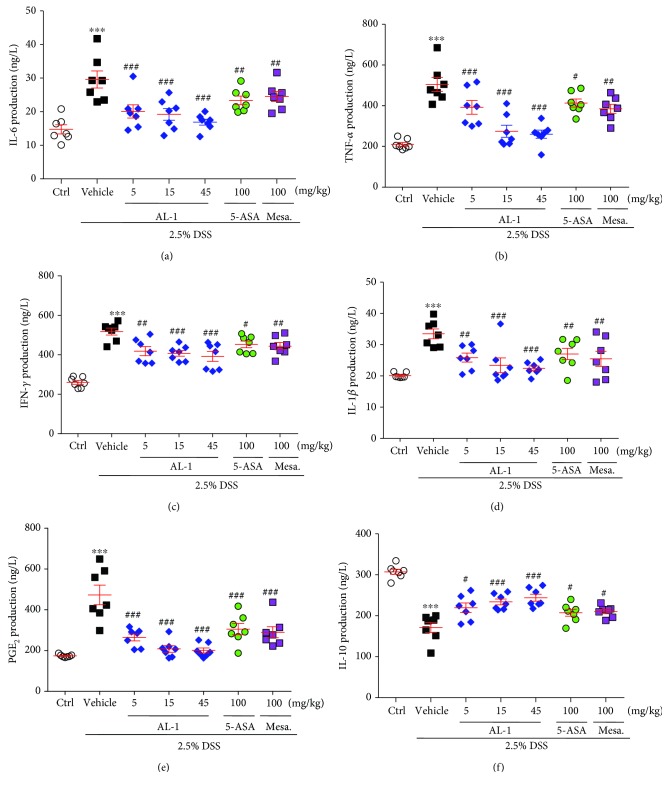
AL-1 reverses the DSS-altered expression of inflammatory cytokines. (a–f) Scattered dot plots showing quantification of cytokine levels measured by ELISA in the sera of C57BL/C mice at day 8 post DSS treatment. Data are presented as the mean ± SEM (*n* = 7 mice/group). ^∗∗∗^*P* < 0.001 compared with the control group and ^#^*P* < 0.05, ^##^*P* < 0.01, and ^###^*P* < 0.001 compared with the vehicle-treated UC model group.

We next examined the levels of cytokines in mouse sera after intestinal injury by DSS. As shown in Figures 4(a)–4(e), compared to the DSS+vehicle group, the production of proinflammatory cytokines IL-1*β*, IL-6, TNF-*α*, IFN-*γ*, and PGE_2_ was markedly decreased in a dose-dependent manner in the sera of mice given AL-1. IL-10 is a well-known anti-inflammatory cytokine [39]. The production of IL-10 was significantly decreased in sera of mice given DSS. However, AL-1 remarkably increased the levels of IL-10 (Figure 4(f)). Treatment with mesalazine or 5-ASA also significantly attenuated the alteration of cytokine expression.

### 3.5. AL-1 Regulates DSS-Induced Activation of NF-*κ*B and MAPK Signaling in Mice

**Figure 5:**
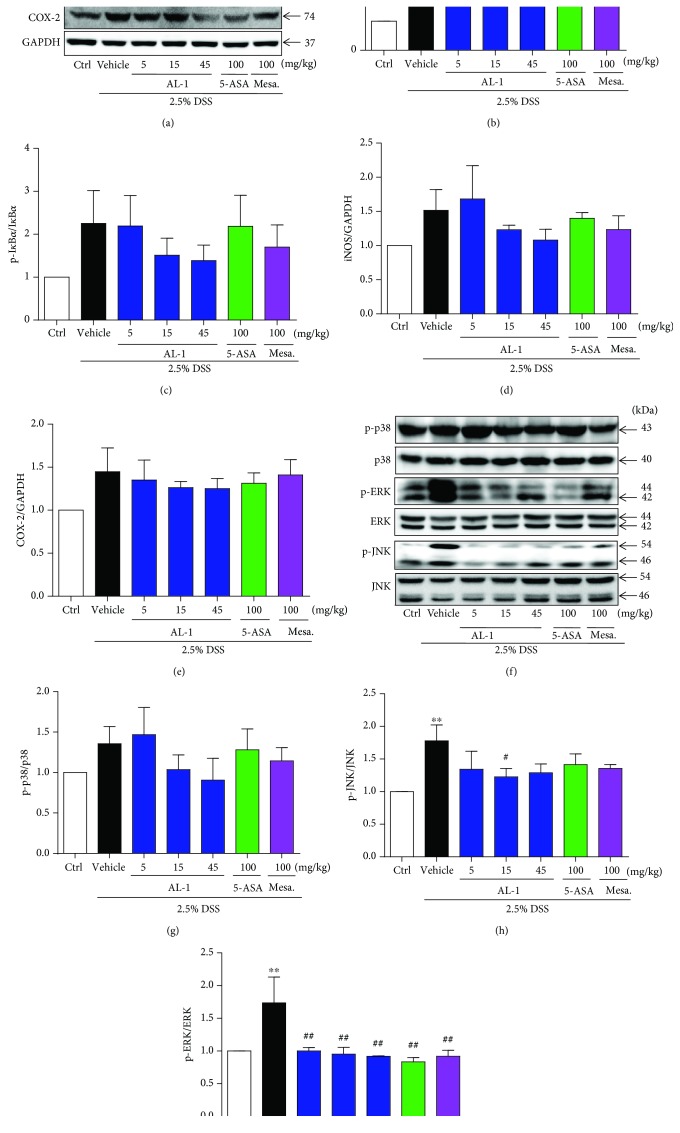
AL-1 inhibits NF-*κ*B and MAPK activation in DSS-induced UC mice. Western blots and related quantification of p-p65, p-I*κ*B*α*, iNOS, COX-2, p-p38, p-ERK, and p-JNK expressed as percent of control. All data are presented as mean values ± SEM for *n* = 3. ^∗∗^*P* < 0.01 and ^∗∗∗^*P* < 0.001 compared with the control group and ^#^*P* < 0.05, ^##^*P* < 0.01, and ^###^*P* < 0.001 compared with the vehicle-treated UC model group.

NF-*κ*B is an important transcriptional regulator of proinflammatory cytokines and mediators [40]. Therefore, we tested the effect of AL-1 on phosphorylation levels of p65 and I*κ*B*α* in colonic tissue homogenates using Western blot analysis. As shown in Figures 5(a)–5(c), compared to the normal control mice, DSS treatment caused activation of NF-*κ*B signaling as evidenced by elevated phosphorylation levels of p65 and I*κ*B*α*. AL-1 treatment markedly reduced phosphorylation of these targeted proteins. The expression of inflammatory mediators (iNOS and COX-2) trended similarly to the activation of NF-*κ*B signaling (Figures 5(a), 5(d), and 5(e)). The MAPK signaling pathway is important for NF-*κ*B activation. As shown in Figures 5(f)–5(i), the expression of phosphorylated p38, JNK, and ERK was significantly upregulated in the colonic tissue of mice with DSS-induced colitis. DSS-stimulated activation of MAPK signals was markedly inhibited by AL-1 in a dose-dependent manner.

### 3.6. AL-1 Reduces ROS and NO Generation Induced by LPS in RAW 264.7 Cells

**Figure 6:**
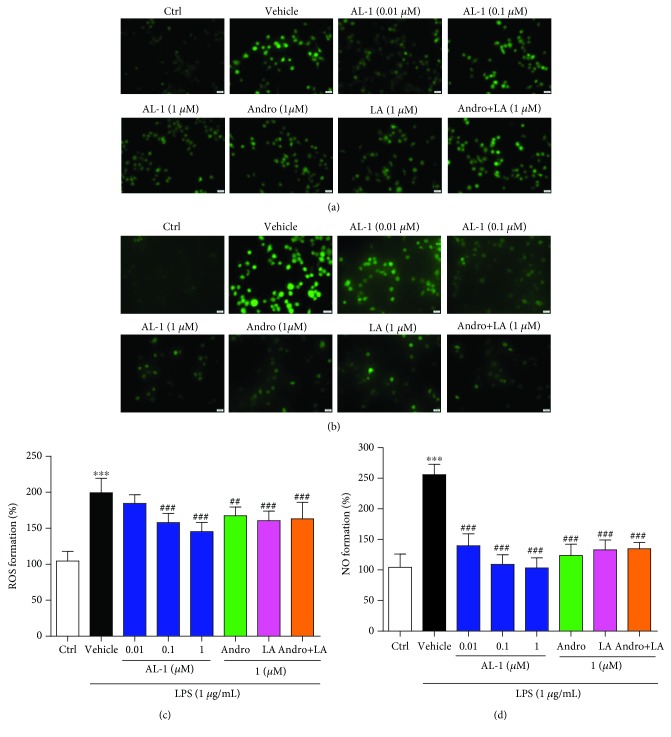
AL-1 represses the generation of ROS and NO in LPS-stimulated RAW 264.7 cells. The fluorescence intensity of ROS was detected using a fluorescence microscope (a) and a BioTek Synergy HT microplate reader (c). The fluorescence intensity of NO was detected using a fluorescence microscope (b) and a BioTek Synergy HT microplate reader (d). Values are expressed as mean ± SEM (*n* = 5‐6 independent experiments). ^∗∗∗^*P* < 0.001 compared with the control group and ^##^*P* < 0.01 and ^###^*P* < 0.001 compared with the model group.

The generation of ROS and NO was elevated significantly in the RAW 264.7 cells treated with LPS (Figure 6). AL-1 decreased the elevated ROS levels by 7.40%, 20.82%, and 27.02% at concentrations of 0.01, 0.1, and 1 *μ*M, respectively (Figures 6(a) and 6(c)). Similarly, AL-1 reduced the elevated NO levels by 45.47%, 57.37%, and 59.59% at concentrations of 0.01, 0.1, and 1 *μ*M, respectively (Figures 6(b) and 6(d)). Interestingly, at an equimolar concentration, AL-1 was more potent in inhibiting the generation of ROS and NO than either of its parent compounds, Andro and LA, as well as their combination.

### 3.7. AL-1 Reduces Proinflammatory Cytokine Production in LPS-Stimulated RAW 264.7 Cells

**Figure 7:**
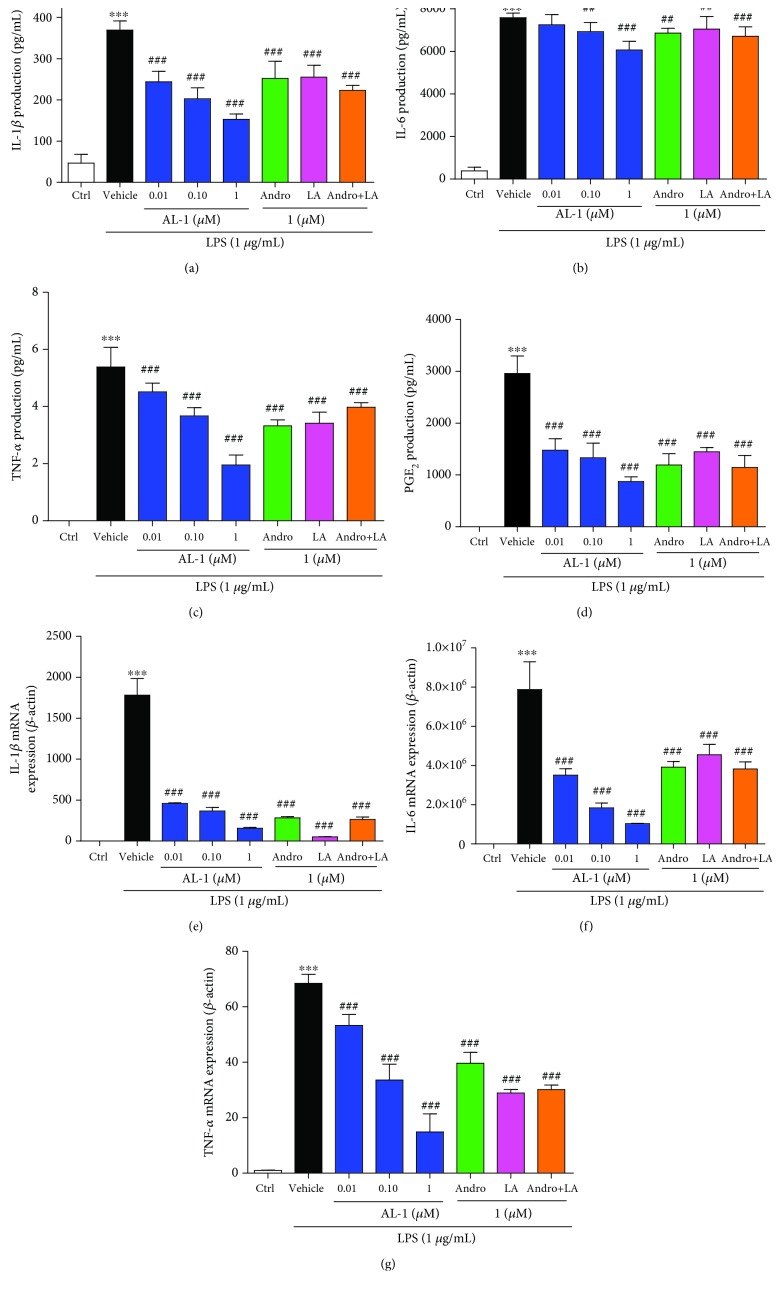
AL-1 reduces proinflammatory cytokine production in LPS-stimulated RAW 264.7 cells. (a–d) RAW 264.7 cells were pretreated with the indicated concentration of AL-1 for 1 h and then stimulated with LPS (1 *μ*g/mL) for 24 h. And the secretion of IL-1*β*, IL-6, TNF-*α*, and PGE_2_ in cell culture supernatants was assessed by ELISA. Values are expressed as mean ± SEM (*n* = 6). ^∗∗∗^*P* < 0.001 compared with the control group and ^#^*P* < 0.05, ^##^*P* < 0.01, and ^###^*P* < 0.001 compared with the vehicle+LPS group. (e–g) The mRNA expression of IL-1*β*, IL-6, and TNF-*α* was determined using quantitative real-time PCR in RAW 264.7 cells. mRNA expression was normalized to *β*-actin expression. Values are expressed as mean ± SEM (*n* = 3 independent experiments). ^∗∗∗^*P* < 0.001 compared with the control group and ^#^*P* < 0.05, ^##^*P* < 0.01, and ^###^*P* < 0.001 compared with the vehicle+LPS group.

To verify our finding that AL-1 inhibited the secretion of IL-1*β*, IL-6, TNF-*α*, and PGE_2_ in vivo, we further tested the effect of AL-1 on cytokine expression in LPS-induced RAW 264.7 cells. As expected, ELISA results revealed that the levels of IL-1*β*, IL-6, TNF-*α*, and PGE_2_ were decreased significantly by AL-1 treatment in a concentration-dependent manner in the supernatant of RAW 264.7 cells stimulated by LPS (Figures 7(a)–7(d)). Moreover, the mRNA expression levels of IL-1*β*, IL-6, and TNF-*α* were markedly decreased after treatment with different concentrations of AL-1 (Figures 7(e)–7(g)).

### 3.8. AL-1 Inhibits the Activation of the NF-*κ*B and MAPK Signaling Pathways in LPS-Induced RAW 264.7 Cells

**Figure 8:**
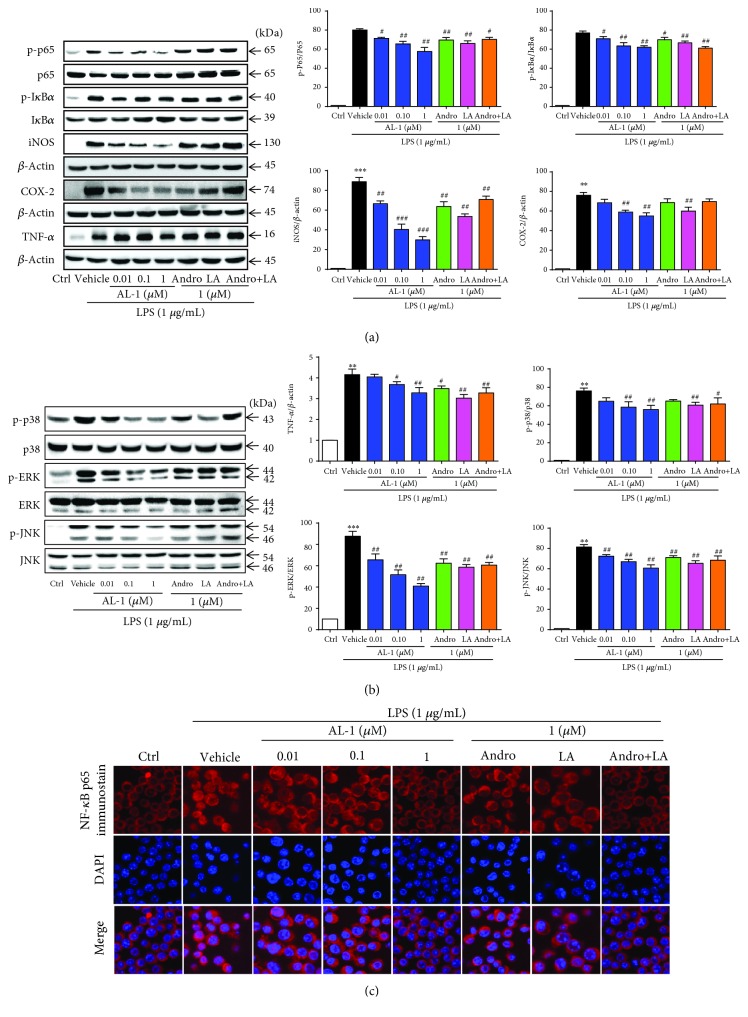
AL-1 attenuates NF-*κ*B and MAPK activation and inhibits the nuclear translocation of NF-*κ*B p65 in LPS-induced RAW 264.7 cells. RAW 264.7 cells were pretreated with the indicated concentration of AL-1 for 1 h and then stimulated with LPS (1 *μ*g/mL) for 24 h. (a) Representative blots showing that AL-1 attenuated the expression of p-65 and p-I*κ*B*α*, iNOS, COX-2, and TNF-*α*. (b) Representative blots showing that AL-1 attenuated the expression of p-p38, p-ERK, and p-JNK. (c) Representative photos showing that coincubation with LPS plus AL-1 inhibited the nuclear translocation of NF-*κ*B p65 in LPS-stimulated RAW 264.7 cells.

AL-1 has been demonstrated to inhibit the activation of the NF-*κ*B and MAPK signaling pathways induced by DSS in mice. We further examined the regulation of AL-1 in these pathways in LPS-induced RAW 264.7 cells. Similar to the findings in vivo, AL-1 treatment markedly reduced the elevated phosphorylation levels of p65 and I*κ*B*α*, as well as the expression levels of iNOS, COX-2, and TNF-*α* (Figure 8(a)). Additionally, the activation of the MAPK signaling pathway and phosphorylation of p38, JNK, and ERK were markedly inhibited by AL-1 in a dose-dependent manner (Figure 8(b)). The nuclear translocation of NF-*κ*B p65 follows I*κ*B*α* phosphorylation degradation. We investigated the nuclear translocation of p65 using immunofluorescent staining in RAW 264.7 cells. As shown in Figure 8(c), LPS significantly stimulated the nuclear translocation of NF-*κ*B p65. However, coincubation with AL-1 effectively blocked the LPS-induced translocation of p65 to the nucleus. AL-1 at 1 *μ*M exhibited the maximum effects in vitro; the parent compounds Andro and LA and their combination at 1 *μ*M were all less potent than AL-1.

## 4. Discussion

**Figure 9:**
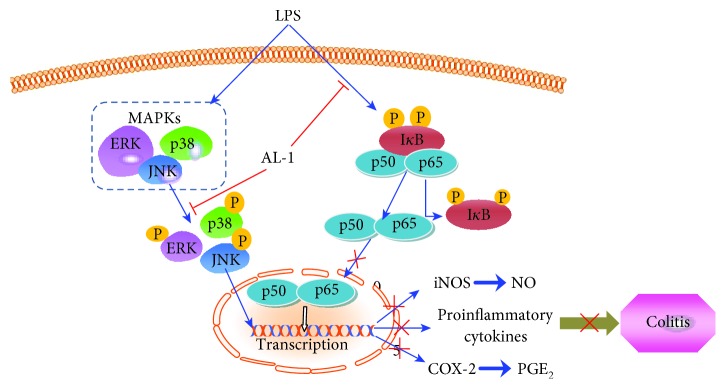
Proposed AL-1 mechanism of action in the mouse model of colitis. AL-1 inhibits both NF-*κ*B and MAPK activation to decrease protein and mRNA levels of proinflammatory cytokines and alleviate colitis.

Because of the pathological features it shares with human IBD and the availability of a quantitative scoring system, the DSS-induced colitis model in mice is now widely used in many studies of human UC. Here, we evaluated the therapeutic effect of AL-1 against experimental DSS-induced colitis in mice. Oral administration of AL-1 suppressed intestinal inflammation as well as attenuated severe mucosal injury and inflammatory symptoms, including body weight loss, colon length shortening, hyperemia, and colonic tissue swelling and ulceration. In addition, AL-1 decreased the accumulation of proinflammatory cytokines in the sera of DSS mice. In LPS-induced RAW 264.7 cells, we showed that AL-1 significantly reduced the generation of ROS and NO, decreased the expression of inflammatory cytokines, and repressed nuclear translocation of NF-*κ*B. Our results demonstrated that AL-1 inhibited the expression of p-I*κ*B*α*, p-p65, and p-MAPKs in mice and in macrophage cells, suggesting that AL-1 prevented DSS-induced ulcerative colitis in mice and LPS-induced inflammation in macrophage cells possibly via downregulation of the NF-*κ*B and MAPK signaling pathways (Figure 9).

MPO, an enzyme produced and released by neutrophils, utilizes and stimulates the production of reactive oxygen species. Intracellular MPO release is of special importance in spreading tissue damage via extracellular matrix inflammation. Accordingly, MPO which serves as a biomarker of neutrophil infiltration can be used to evaluate UC disease severity [41]. iNOS and COX-2 are enzymes for NO and PGE_2_ synthesis, respectively. They are involved in the injured mucosa and submucosa of the intestine [42]. Both iNOS and COX-2 can thus be considered indices of inflammatory damage. In the present study, treatment with AL-1 decreased the increase in MPO, iNOS, and COX-2 activities induced by DSS in mice. Consistent with these findings, AL-1 also reduced the elevated levels of iNOS, COX-2, NO, and PGE_2_ in cultured macrophage cells. Histopathological analysis showed abnormal crypts, crypt loss, mucosal inflammatory cell infiltration, and submucosal congestion and edema in DSS-induced mice. Our findings suggested that AL-1 could reverse the infiltration of inflammatory cells into colonic tissues and protect against DSS-induced colitis and LPS-induced inflammation by inhibiting the activities of iNOS and COX-2.

It is worth noting that high levels of proinflammatory cytokines are a hallmark of DSS-induced colitis. Proinflammatory cytokines can amplify the inflammatory cascade. Furthermore, it can result in tissue damage by increasing the production of destructive enzymes and free radicals [43]. Oral administration of AL-1 significantly attenuates the production of IL-1*β*, IL-6, TNF-*α*, PGE_2_, and IFN-*γ* in the sera of colitis mice. Importantly, AL-1 produced similar inhibitory effects on the production of proinflammatory cytokines in LPS-induced macrophage cells. Interleukin-10 (IL-10) is an important cytokine with anti-inflammatory properties, which inhibits the production of proinflammatory cytokines by activated macrophages [43, 44]. In our study, we found that AL-1 increased the secretion of IL-10 dramatically in sera of DSS-induced colitis mice. We can thus conclude that AL-1 ameliorates colitis by modulating the balance of pro- and anti-inflammatory cytokines.

We demonstrated that AL-1 decreased the production of the proinflammatory cytokine. NF-*κ*B is a ubiquitous transcription factor that governs the expression of genes encoding inflammatory cytokines [45]. NF-*κ*B maintains I*κ*B*α* in an inactive form in the cytoplasm of unstimulated cells. Once activated, NF-*κ*B translocates to the nucleus via the phosphorylation and rapid degradation of I*κ*B*α* [46, 47]. After nuclear translocation of its cytoplasmic complexes, activated NF-*κ*B promotes the expression of various inflammatory genes (iNOS, COX-2, and TNF-*α*), including inflammatory cytokines known to be involved in UC [46, 48]. The phosphorylation of p65 and I*κ*B*α* plays a key role in regulation of the NF-*κ*B pathway. To investigate whether the inhibitory effect of AL-1 on the expression of inflammatory mediators is associated with activity of the NF-*κ*B pathway, we measured the effect of AL-1 on NF-*κ*B activation by analyzing the translocation of p65 to the nucleus and the phosphorylation of p65 and I*κ*B*α*. Our previous studies have found that AL-1 significantly reduced the phosphorylation of p65 and I*κ*B*α* proteins in TNBS-induced colitis mice [26]. On this matter, we asked whether the protective effect of AL-1 is due to the inhibition of the NF-*κ*B signaling pathway in DSS-induced colitis mice. In this study, we found that AL-1 inhibits the phosphorylation of p65 and I*κ*B*α* in DSS-induced colitis mice. Consistent with the results in vivo, AL-1 also inhibited the phosphorylation of p65 and I*κ*B*α* and prevented nuclear translocation of p65 in LPS-stimulated RAW 264.7 cells. The downregulation of NF-*κ*B has powerful therapeutic potential in DSS-induced murine colitis as reported previously [49]. Thus, it can be inferred that inhibition of NF-*κ*B activation may explain at least some of the beneficial effects provided by AL-1 in UC.

MAPKs are the signaling molecules and enzymes upstream of NF-*κ*B. Similar to NF-*κ*B signaling, MAPK activation has been commonly observed in inflammatory responses [50]. Inflammatory stimuli can activate the MAPK signaling pathway, and MAPKs are implicated in regulating the production of proinflammatory cytokines TNF-*α*, IL-1*β*, and IL-6 as well as the production of the anti-inflammatory cytokine IL-10 [46]. Previous studies showed that the activity of MAPKs was significantly increased during IBD intestinal epithelial injury. Tissue immunohistochemistry revealed high expression levels of p38 MAPK in macrophages and neutrophils of the intestinal lamina propria [51]. Compared with normal people, the expression of p-p38 MAPK was significantly higher in UC patients and showed a positive correlation with the degree of ulcerative colitis [52]. Accumulating evidence suggests that the MAPK signaling pathway can affect the balance of inflammatory cytokines to preserve the health of the gastrointestinal tract, thus influencing the inflammatory process. In this study, we found that the phosphorylation of p38, JNK, and ERK was inhibited by AL-1 both in vivo and in vitro. These results demonstrated that the anti-inflammatory activity of AL-1 is probably mediated at least in part by inactivation of MAPK.

## 5. Conclusion

In conclusion, the present work demonstrates that AL-1 suppressed the inflammatory responses in RAW 264.7 macrophages and in an experimental animal model of UC, probably through the inhibition of NF-*κ*B and MAPK signaling pathways. Inhibition of NF-*κ*B and MAPK signaling pathways might be a promising therapeutic strategy for the treatment of UC.

## Figures and Tables

**Table 1:** Specific primers used in real-time PCR analysis.

Genes	Primer	Sequence (5′→3′)
IL-1*β*	FW	TCCAGGATGAGGACATGAGCAC
	RV	GAACGTCACACACCAGCAGGTTA
IL-6	FW	CCACTTCACAAGTCGGAGGCTTA
	RV	CCAGTTTGGTAGCATCCATCATTTC
TNF-*α*	FW	TATGGCCCAGACCCTCACA
	RV	GGAGTAGACAAGGTACAACCCATC
*β*-Actin	FW	CATCCGTAAAGACCTCTATGCCAAC
	RV	ATGGAGCCACCGATCCACA

Note: IL-6: interleukin-6; IL-1*β*: interleukin-1*β*, TNF-*α*: tumor necrosis factor-*α*; FR: forward; RV: reverse.

## Data Availability

The data used to support the findings of this study are available from the corresponding authors upon request.
